# Identification and validation of cuproptosis related genes and signature markers in bronchopulmonary dysplasia disease using bioinformatics analysis and machine learning

**DOI:** 10.1186/s12911-023-02163-x

**Published:** 2023-04-14

**Authors:** Mingxuan Jia, Jieyi Li, Jingying Zhang, Ningjing Wei, Yating Yin, Hui Chen, Shixing Yan, Yong Wang

**Affiliations:** 1 Adcote School Shanghai, Shanghai, 200000 China; 2 Shanghai Literature Institute of Traditional Chinese Medicine, Shanghai, 200000 China; 3 ChengZheng Wisdom (Shanghai) Health Sciences and Technology Co., Ltd, Shanghai, 200000 China; 4 Shanghai Daosh Medical Technology Co., Ltd, Shanghai, 200000 China

**Keywords:** Bronchopulmonary dysplasia disease, Cuproptosis, Machine learning, Biomarkers, Bioinformatics analysis

## Abstract

**Background:**

Bronchopulmonary Dysplasia (BPD) has a high incidence and affects the health of preterm infants. Cuproptosis is a novel form of cell death, but its mechanism of action in the disease is not yet clear. Machine learning, the latest tool for the analysis of biological samples, is still relatively rarely used for in-depth analysis and prediction of diseases.

**Methods and results:**

First, the differential expression of cuproptosis-related genes (CRGs) in the GSE108754 dataset was extracted and the heat map showed that the expression of NFE2L2 gene was significantly higher in the control group whereas the expression of GLS gene was significantly higher in the treatment group. Chromosome location analysis showed that both the genes were positively correlated and associated with chromosome 2. The results of immune infiltration and immune cell differential analysis showed differences in the four immune cells, significantly in Monocytes cells. Five new pathways were analyzed through two subgroups based on consistent clustering of CRG expression. Weighted correlation network analysis (WGCNA) set the screening condition to the top 25% to obtain the disease signature genes. Four machine learning algorithms: Generalized Linear Models (GLM), Random Forest (RF), Support Vector Machine (SVM), and Extreme Gradient Boosting (XGB) were used to screen the disease signature genes, and the final five marker genes for disease prediction. The models constructed by GLM method were proved to be more accurate in the validation of two datasets, GSE190215 and GSE188944.

**Conclusion:**

We eventually identified two copper death-associated genes, NFE2L2 and GLS. A machine learning model-GLM was constructed to predict the prevalence of BPD disease, and five disease signature genes NFATC3, ERMN, PLA2G4A, MTMR9LP and LOC440700 were identified. These genes that were bioinformatics analyzed could be potential targets for identifying BPD disease and treatment.

## Introduction

Bronchopulmonary Dysplasia (BPD) is a disease with a high prevalence in preterm infants, affecting 35% of all babies born prematurely each year [1]. The disease is caused by a number of factors [2, 3], such as the weight and survival of the preterm infant [4, 5]. Because the lungs of preterm infants are at an immature stage, inappropriate treatment can impair lung growth and produce structural changes in the affected lungs due to reduced alveoli and disturbed matrix remodeling, which may persist into adolescence [6]. Current research findings suggest that treatment options for BPD are limited to supportive care such as hyperoxia and medications [1, 7], and that relatively advanced screening and diagnostic imaging techniques are only available for specific populations [8], and that more efficient and feasible treatments need to be developed.

In contrast to the established apoptotic modalities, Cuproptosis is a novel way of causing cell death through the accumulation of copper ion concentrations in cells [9]. Available studies suggest that the specific mechanism of action of Cuproptosis is the induction of cell death by targeting lipid acylated TCA cyclins [10]. The combined analysis of Cuproptosis and disease has focused on oncology, such as bladder cancer, liver cancer and melanoma [11–14], involving multiple aspects of tumor microenvironment, clinical outcome and patient prognosis. Fewer studies have been performed in non-oncology areas, with results published only in rheumatoid arthritis, inflammatory bowel disease and Alzheimer’s disease [15–17]. Through the published literature, non-tumor diseases are less studied for Cuproptosis and need to be studied in more depth.

Although machine learning models are a type of technology derived from artificial intelligence research, they have gradually taken an important place in the analysis of large amounts of complex biological data in recent years [18] and have been successfully applied in disease diagnosis, drug screening and basic research [19]. In the field of protein function, the incorporation of machine learning into analytical models can improve the accuracy of prediction and in-depth analysis of protein function [20]. In the field of metabolic engineering, machine learning has improved data analysis methods, saving time and improving the accuracy of predicting metabolic results [21]. Generalized Linear Models (GLM) is a regression model for non-normal dependent variables [22]; Random Forest (RF) can evaluate the importance of variables and model predictions [23]; Support Vector Machine (SVM) is a two-class classification model that assigns labels to objects through instance learning [24]; and Extreme Gradient Boosting (XGB) is to integrate the prediction results of multiple classifiers as the most do that prediction [25]. It is a new attempt to apply the above machine learning algorithm model methods to disease target gene analysis and feature gene prediction.

In summary, in this study, we used BPD disease as an entry point to explore Cuproptosis genes expression in disease transcriptome cohorts, the location of significantly expressed genes in human chromosomes and immune cell differences. Consistent clustering analysis identifies unique pathways across different subgroups. WGCNA combines four machine learning models to mine genes characteristic of BPD disease, evaluates model performance and sets up a validation group to test model accuracy.

## Methods and materials

### Data sources and analysis tools

High throughput gene expression data for human were retrieved from the Gene Expression Omnibus data base (GEO) using the search term “Bronchopulmonary Dysplasia” [26]. All studies involving BPD disease were screened for the following inclusion criteria: (1) BPD infant cord blood. (2) Provide specific study platform and technical information. (3) Normal infant cord blood was included in each dataset as a control group. The sequencing cohort used for the analysis is based on the GPL13497 platform GSE108754 samples from the GEO. The validation dataset is from the same database, GSE190215 based on GPL30862 platform and GSE188944 based on GPL14951 platform. The bioinformatics analysis tools involve text fast processing of Perl language [27] scripts, and the compiler is Strawberry perl (version 5.30.0.1). Systematic analysis and visualization were performed using R language scripts (R version 4.1.3) [28], which contains a number of data analysis packages. When P value was used as a test for significance of difference, P less than 0.05 was statistically significant.

### Expression of CRGs

The expression matrix of BPD disease genes was obtained using perl language script, and the expression of copper death genes in the matrix was extracted from the corrected data to obtain the expression matrix of 19 CRGs [29]. Based on the CRGs expression matrix, the “limma” package [30] was used to analyze the correlation between differential expression and CRGs between the premature birth of infants with BPD disease (treat) and healthy preterm infants (control) groups. The “RCircos” package [31] was used to annotate the distribution of CRGs on chromosomes.

### Immune cell related expression analysis

The tumor immune infiltration analysis package “CIBERSORT” [32] was introduced to observe the expression of immune cells in the control and treat groups, and box plots can show the differences in immune cells between the different groups.

### Consistency clustering analysis

Samples were grouped into different subtypes based on the expression of differential CRGs in the samples. The “ConsensusClusterPlus” [33], an R package specifically designed for onsistency clustering analysis, was used to analyze only the experimental group samples and set the clustering K values. The most reliable results of the clustering analysis were obtained by judging the cumulative distribution function of K taking different values. Immunocytic infiltration analysis is performed on the typed groups. The Gene Set Variation Analysis (GSVA) uses two data packages “GSEABase” and “GSVA” to construct functions and set parameters to evaluate whether different metabolic pathways are enriched among samples without typing.

### WGCNA method to construct gene co-expression network

The data package “WGCNA” [34], which is required for weighted correlation network analysis (WGCNA) built into the R language, was used to select the top 25% of the most fluctuating genes in the BPD disease gene expression matrix for analysis. After first removing the offending genes and samples from the data, the samples were clustered. Then, the Power value power index range of 1:20 was set and the scatter plot showed the fit index and average connectivity. Finally, the genes are clustered and the dynamic modules identify the modules where the genes are located and the modules are clustered. The similarity between modules is found and the module with the smallest p-value of the correlation test is identified as the disease key gene module.

### Machine learning model construction

Four R language packets “caret” [35], “dalex”, “randomForest” [36] and “xgboost” [37] are combined to build four machine learning models: Generalized Linear Models (GLM), Random Forest (RF), Support Vector Machine (SVM), and Extreme Gradient Boosting (XGB). The “kernlab” [38] package has a built-in cluster of algorithms that can perform many tasks in machine learning. The expression of core genes in the intersection of WGCNA is extracted and the results are predicted using four models. The accuracy of the models is evaluated by plotting the residual box line, the cumulative distribution of residual directions, and the ROC curves of the models. Importance scores are assigned to each model gene to filter out the characteristic genes for BPD disease.

### Machine learning model validation

The disease signature gene expression obtained from the machine learning model with the highest accuracy is extracted, the Nomogram is plotted to score each signature gene, and the probability of the patient developing the disease is finally assessed based on the combined score. The calibration curve and decision curve can reflect the accuracy of the Nomogram plot scoring mechanism. The expressions of the feature genes are learned according to the construction machine, validated on two datasets, GSE190215 and GSE188944, and ROC curves are drawn to demonstrate the prediction results.

## Results

### Expression of CRGs in BPD

**Fig. 1:**
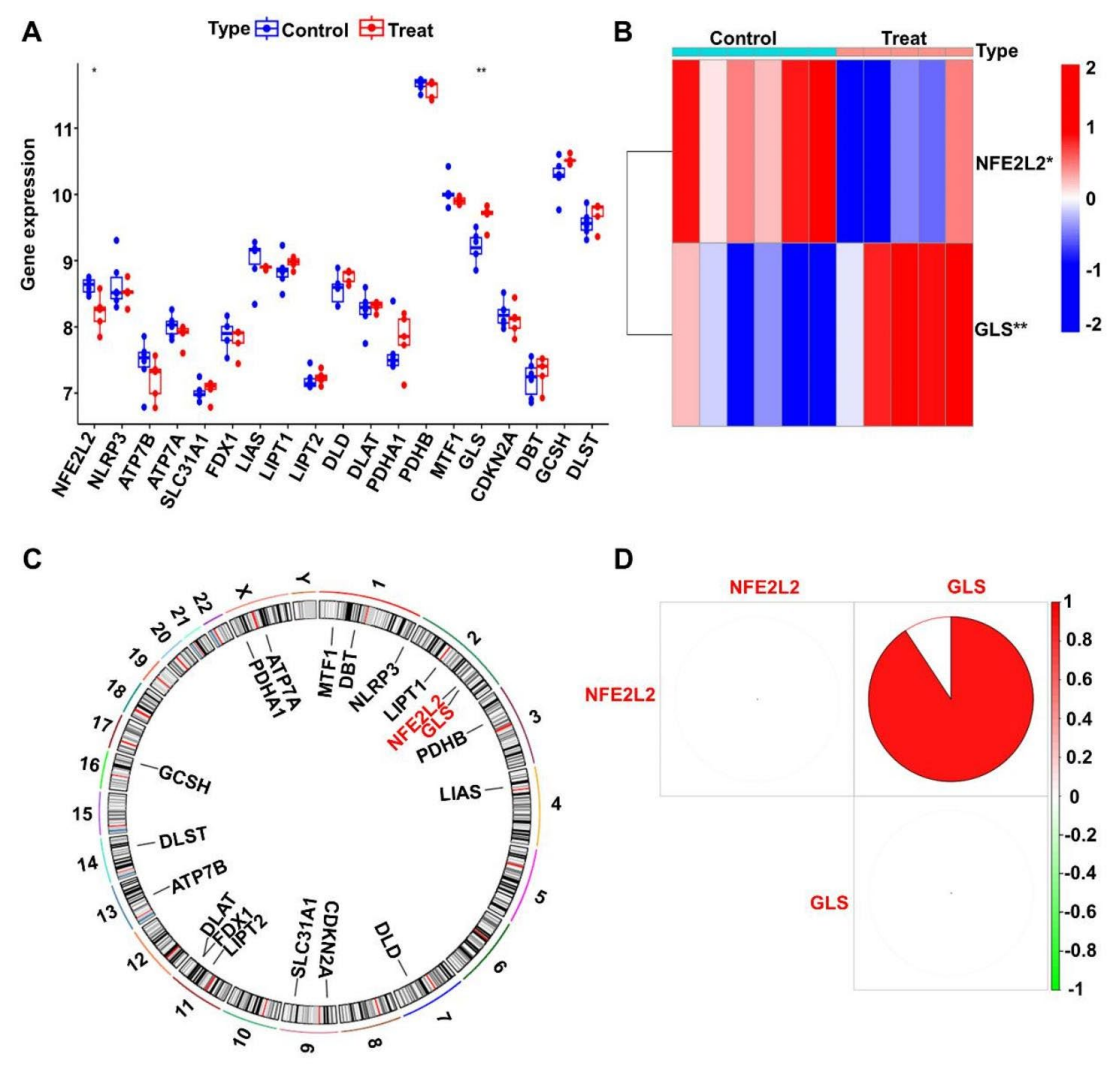
Expression of cuproptosis-related genes (CRGs) in bronchopulmonary dysplasia (BPD) disease. (**A**) Differentially expressed of CRGs. ***p < 0.001,**p < 0.01 and *p < 0.05. (**B**) Heat map of significantly different CRGs expression between different subgroups. ***p < 0.001,**p < 0.01 and *p < 0.05. (**C**) The position of CRGs on 23 chromosomes. (**D**) The correlation of CRGs in BPD disease

The list of CRGs contains 19 genes, and it can be seen in the differential expression analysis plot (Fig. 1A) that two genes, NFE2L2 and GLS, are differentially expressed in the Control and Treat groups. As seen in the heat map (Fig. 1B), for the two CRGs that were significantly differentially expressed, NFE2L2 was upregulated in the Control group and downregulated in the Treat group. However, GLS is opposite to this, which is worth our attention. On the circle plot of gene distribution on chromosomes (Fig. 1C), two genes are mainly associated with human chromosome 2. In terms of gene correlation (Fig. 1D), the two genes show a positive correlation.

### CRGs and immune cell correlation analysis

**Fig. 2:**
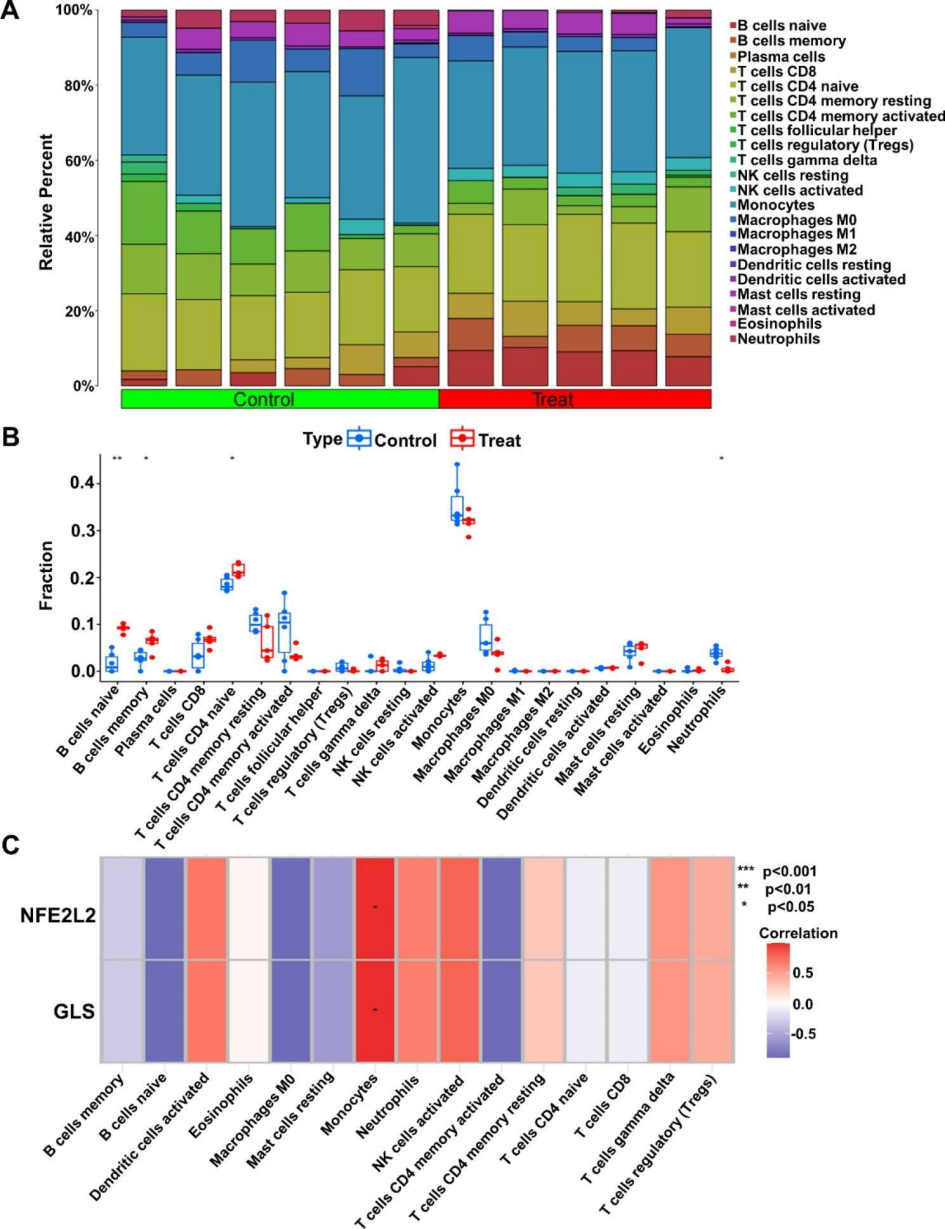
Cuproptosis-related genes (CRGs) and immune cell correlation analysis. (**A**) Histogram of the expression levels of 22 immunocyte subgroups in control and treat groups. (**B**) The expression differences of immunocytes in different groups. ***p < 0.001,**p < 0.01 and *p < 0.05. (**C**) The expression of 2 CRGs between 15 immunocyte subgroups

From the histogram of 22 immune cell infiltration in different groups (Fig. 2A), it can be seen that the Treat group had higher content of B cells naive and B cells memory, while the Control group had higher content of T cells CD4 naive and Neutrophils cells, and the content of other immune cells differed less, the specific reasons need further experimental verification. In the immune cell differential analysis plot (Fig. 2B), the conclusions obtained were consistent with the infiltration results, with statistically significant differences in B cells naïve, B cells memory, T cells CD4 naive and Neutrophils between the different subgroups (p < 0.05). Analysis of the correlation between differentially expressed CRGs and immune cells in BPD disease showed (Fig. 2C) that 15 immune cells were positively regulated with CRGs, and negative regulation was mainly reflected in B cells naive, Macrophages M0, and T cells CD4 memory activated. The most significant correlation in the positive regulatory relationship was found in Monocytes cells.

### Classification of DCRGs into two subtypes by consistency clustering

**Fig. 3:**
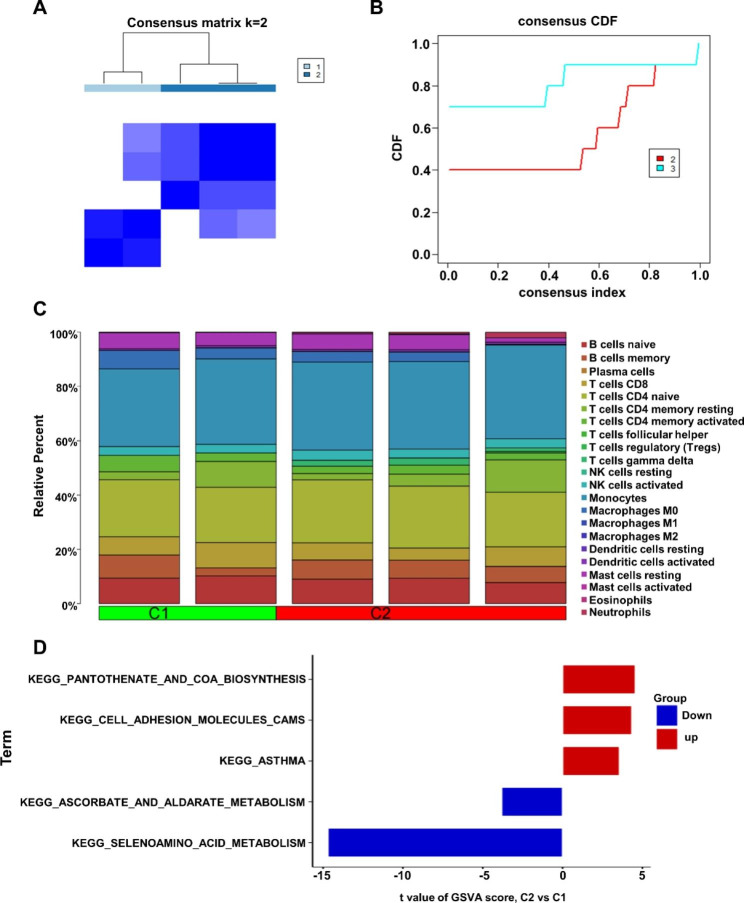
The expression of DCRGs was divided by consistent clustering into two different subtype samples and biological characteristics. (**A**) Consensus matrix heat map defining two clusters (k = 2) and their correlation area. (**B**) Consistency cumulative distribution function curve. (**C**) The abundance of each TME infiltrating cell in two clusters. (**D**) GSVA of biological pathways between two distinct subtypes, where red and blue represent up- and down-regulated pathways, respectively

The BPD samples were typed based on the expression of differential CRGs genes. The two clustering subgroups defined by the consistency matrix heat map (Fig. 3A) and the consistency cumulative distribution function (CDF) curve (Fig. 3B) demonstrate that the CDF reaches an approximate maximum at K = 2, when the clustering results are most reliable. Looking at the immune cell content between subgroups C1 and C2, the histogram (Fig. 3C) shows less variability in immune cells between the two groups. The GSVA analysis plot (Fig. 3D) can be observed that there are five pathways that are differential between the different subtypes. Three pathways: pantothenate and coa biosynthesis, cell adhesion molecules cams, and asthma are shown in red, representing positive regulatory relationships in the C2 subgroup; ascorbate and aldarate metabolism, and selenoamino acid metabolism are shown in blue, representing the positive regulatory relationship in the C1 subgroup.

### Application of WGCNA to construct a gene co-expression network in BPD Patients

**Fig. 4:**
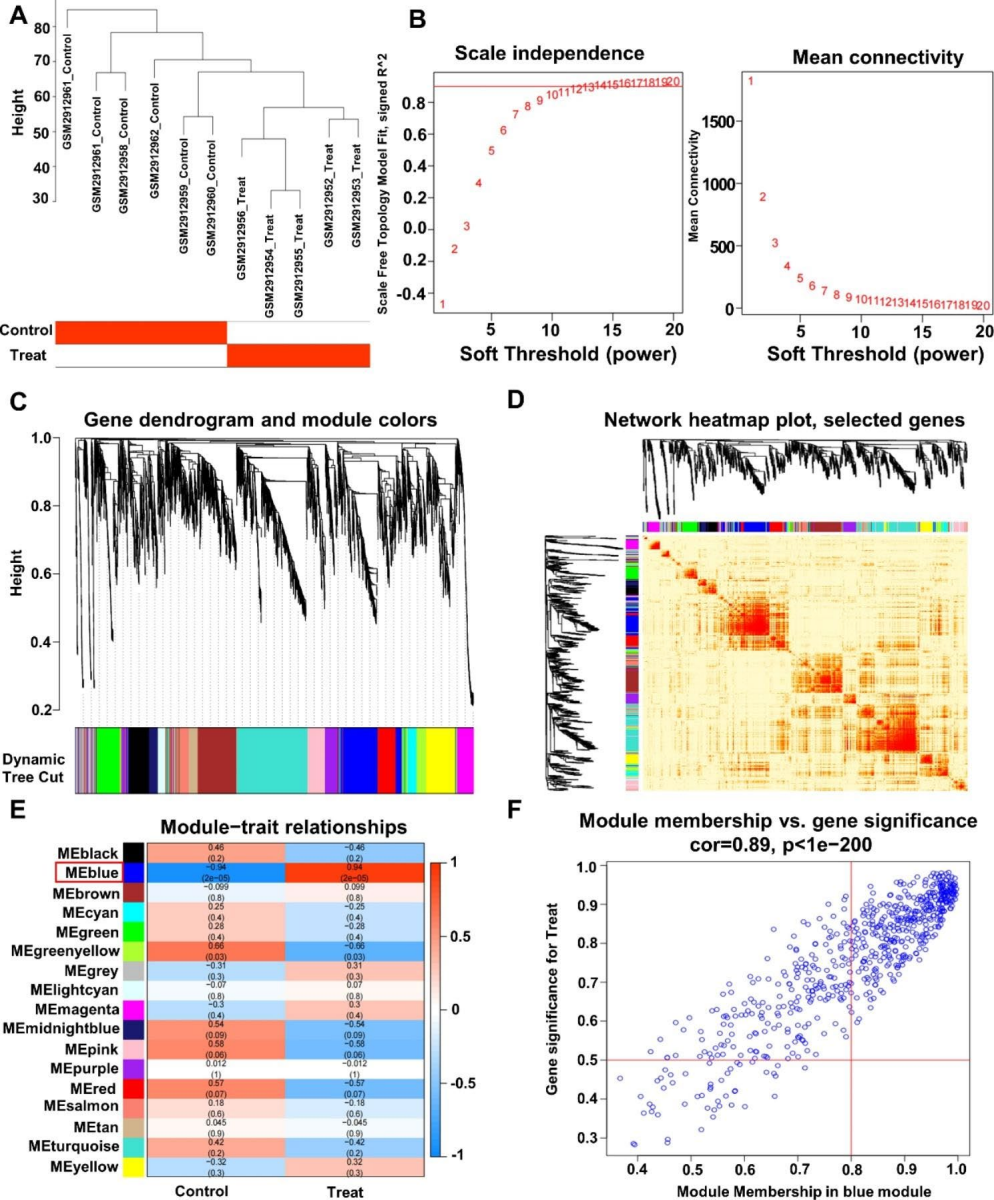
Application of WGCNA to construct a gene co-expression network in BPD patients. (**A**) A weighted co-expression network. (**B**) Scale independence and mean connectivity. (**C**) Gene dendrogram and modules before merging. (**D**) Visualizing the gene network using a heat map plot. (**E**) Pearson correlation analysis of merged modules and CAF score. (**F**) Scatterplot of MM and GS from the blue module

WGCNA first clustered the samples (Fig. 4A) and used the top 20% of the BPD disease samples with the most divergent genes for analysis, setting a soft threshold of 2 (Fig. 4B). The gene expression matrix was then dynamically identified in modules, each containing no less than 100 genes (Fig. 4C). The module gene correlation model was constructed (Fig. 4D), and the darker the color at the connection of two modules, the stronger the correlation. A total of 17 co-expression modules were aggregated in the cohort (Fig. 4E), and the blue module had the strongest negative correlation with the Control group score (Cor = -0.94, P = 2e-05) and the strongest positive correlation with the Treat group score (Cor = 0.94, P = 2e-05). Finally, setting the gene importance greater than 0.5 and the gene-module correlation greater than 0.8, 482 pivotal genes were screened as potential BPD-related genes from the 643 gene force of the blue module (Fig. 4F).

### Building a machine learning model to identify BPD disease signature genes

**Fig. 5:**
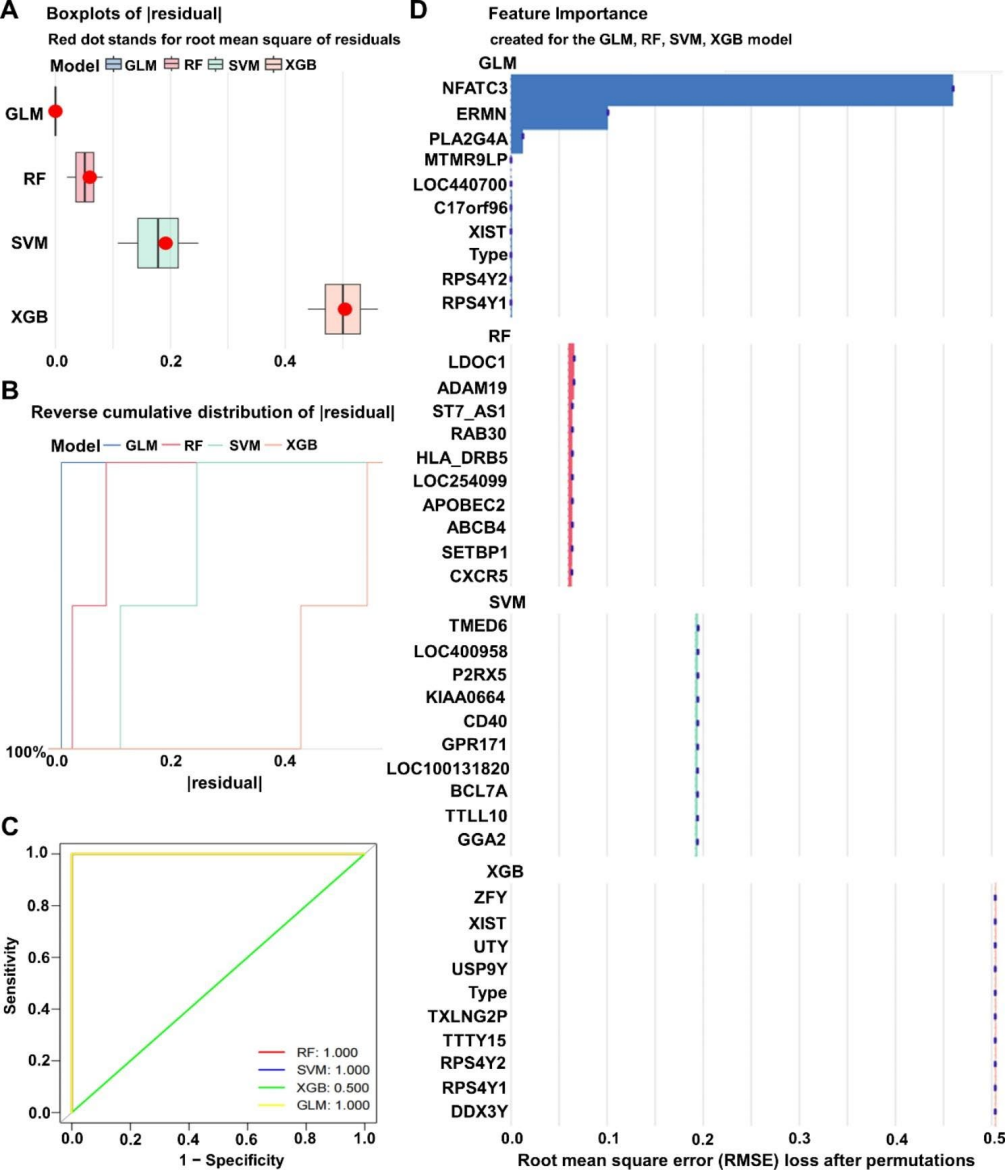
Building a machine learning model to identify BPD disease signature genes. (**A**) Four machine learning algorithms residual box line diagram. (**B**) Reverse cumulative distribution of residual among different machine learning models. (**C**) ROC curve to verify the accuracy of the model. (**D**) Importance score of feature genes in the model

The 482 pivotal gene expression profiles from the WGCNA blue co-expression module were used to construct prediction functions using four machine learning models. In the residual box line plot of the models (Fig. 5A), the red dots represent the root mean square of the residuals, GLM has the smallest value of residuals, XGB has the largest value of residuals, and the distribution of residuals of RF and SVM is between 0 and 0.2. The conclusion obtained from the plot of the reverse cumulative distribution of residuals (Fig. 5B) is consistent with the above results. The results of the ROC curve (Fig. 5C) showed that the area under the curve of the XGB model was 0.5, and the area of the remaining three models was 1. From the results, it can be seen that the XGB model has a smaller curve area with other machine learning models, which may be related to the overfitting of the model. The importance analysis of genes was performed for the four methods, and the gene importance scores of the four methods were obtained (Fig. 5D). In the GLM model, the top five genes with the highest importance scores were NFATC3, ERMN, PLA2G4A, MTMR9LP and LOC440700; LDOC1, ADAM19, ST7_AS1, RAB30 and HLA_DRB5 are the most important genes in the RF model; TMED6, LOC400958, P2RX5, KIAA0664 and CD40 occupy an important position in the SVM model; the root mean square error (RMSE) loss after traversal for ZFY, XIST, UTY, USP9Y and Type importance scores is 0.5 in the XGB model. In summary, we choose the machine learning model GLM with the highest accuracy.

### Machine learning model prediction accuracy check

**Fig. 6:**
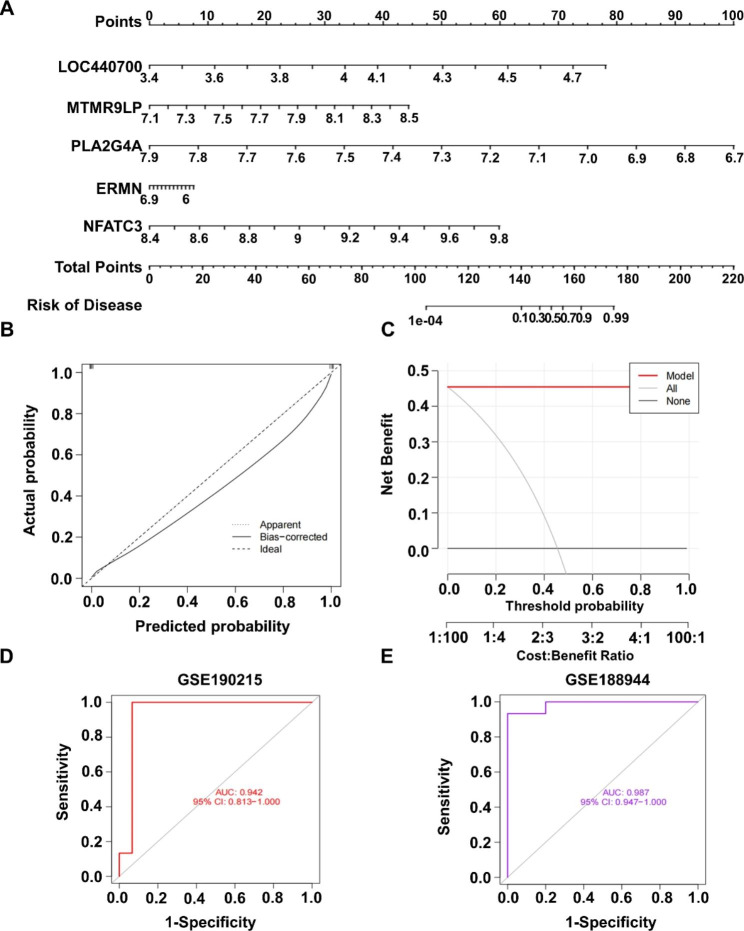
Machine learning model prediction accuracy check. (**A**) Nomogram showing prediction of BPD prevalence using signature genes. (**B**) Calibration curve illustrating the accuracy of nomogram. (**C**) Decision curve illustrating the accuracy of the model. (**D**) Validate model accuracy on the GSE190215. (**E**) Validate model accuracy on the GSE188944.

The first five genes of the GLM model were selected as the disease signature genes, and the nomogram (Fig. 6A) was constructed to predict the incidence of BPD disease. Each signature gene would have a separate score interval, and the scores of all genes were summed to obtain the final score and then compared to the incidence rate. The predictive power of the nomogram is demonstrated using the calibration curve (Fig. 6B), where the solid and dashed lines are closer to each other in the figure, indicating the high accuracy of the model. The Model represented by the red line in the decision curve (Fig. 6C) is far away from the all curve, again indicating the model effect. Two validation datasets, GSE190215 and GSE188944, were set up to identify the models (Fig. 6D, E), and surprisingly, the accuracy of our constructed models can reach 94.2% and 98.7%.

## Discussion

In this study, we obtained CRGs based on BPD disease, combined with Cuproptosis and machine learning to analyze high-throughput sequencing group data. The copper death genes NFE2L2 and GLS, which were differentially expressed in the disease and healthy groups, were obtained. Coincidentally, both genes were on chromosome 2 and both genes showed positive correlation; and both genes were associated with Monocytes cells, again in a positive regulatory relationship. Consistent clustering of the samples by expression of CRGs showed that they could be clustered into C1 and C2 subgroups. GSVA analysis of the pathways of both subtypes showed that the subgroup C1 has 2 unique pathways relative to subgroup C2, and subgroup C2 has 3 pathways. WGCNA found hidden BPD potential genes in the blue module. GLM possessed higher accuracy among the four machine learning models and established five disease marker genes.

The first two genes identified were Cuproptosis-associated genes. The NFE2L2 gene is now mostly studied in the cancer field; in esophageal squamous cell carcinoma NFE2L2 may confer oncogenic activity [39]; in cervical squamous carcinoma it is involved in immune prognosis, mainly acting in the tumor microenvironment [40]; autophagy and the NFE2L2 pathway activate ubiquitin ligases in prostate cancer [41]. In the non-cancerous cellular domain, NFE2L2 gene variants affect metabolic and renal function parameters in patients with diabetes and hypertension [42]; are also genetic markers of susceptibility to cirrhosis [43] ; and evidence has even been found in the effect of obesity on heart rate [44]. It has been demonstrated that GLS is an anti-cuproptosis gene [45]. GLS has been identified as a genetic marker for the diagnosis of acute myocardial infarction [46] and has been less studied in other diseases, but results have been published in cancers such as glioma [47], breast cancer [48] and liver cancer [49].However, these two marker genes have not been studied in the subject of prematurity. It is worth our attention that these genes are related to human chromosome 2, which provides a direction for future genetic screening of embryos. Also, there is a potential link between the genes and immune cells.

Adding machine learning algorithms in artificial intelligence to statistical analysis in biology has good predictive effect. For the data in this study, the GLM model had the highest composite score in the prediction of BPD disease signature genes. Five disease signature genes (NFATC3, ERMN, PLA2G4A, MTMR9LP and LOC440700) were screened and scored for a range of values for different genes to assess the probability of preterm infants with the disease. NFATC3 gene can inhibit or enhance cancer progression by inducing and modulating other pathways [50–52]. ERMN genes are mostly present in the expression profile of autistic patients [53, 54]. The PLA2G4A gene has been shown to be associated with childhood asthma [55]. The MTMR9LP gene and LOC440700 gene are both long non-coding RNAs. The MTMR9LP gene may be a marker for the treatment and prevention of bisphosphonate-induced osteonecrosis of the jaw [56], and the mechanism of LOC440700 gene has not been identified. It can be seen that none of the above five genes selected by the GLM model have been studied in BPD disease. The five disease marker genes have the potential to be marker genes for BPD disease prevention, prediction and treatment. They can help obstetricians to evaluate and treat preterm babies appropriately. Of course, our study is only at the stage of data analysis, and further basic medical experiments are needed to support the results of the research process.

## Conclusions

In the present study, we identified two differentially expressed Cuproptosis-associated genes, NFE2L2 and GLS, in BPD disease. Based on these two genes, we explored the immune signature and immune correlated expression. Combined with WGCNA analysis and machine learning models to screen for disease signature genes, the GLM model was identified as a predictive model for BPD disease. And five disease signature genes were predicted, NFATC3, ERMN, PLA2G4A, MTMR9LP and LOC440700. We introduced machine learning algorithms in artificial intelligence to statistical analysis in biology, adding to the big data analysis in medicine.

## Data Availability

All analysis data in this article are from publicly available databases. Users can download relevant data for free for research and publish relevant articles. (https://www.ncbi.nlm.nih.gov/geo/query/acc.cgi?acc=GSE108754)
